# Integrated CT-based assessment of muscle and adiposity for risk stratification in advanced pancreatobiliary cancer

**DOI:** 10.3389/fnut.2026.1792814

**Published:** 2026-05-14

**Authors:** Se Eung Oh, Sang Uk Han, Ye Seul Seong, Yoo Eun Yoon, Yeon Gil Jeong, Ji Soo Park, Ik Jae Lee, Hei-Cheul Jeung

**Affiliations:** 1 Division of Medical Oncology, Department of Internal Medicine, Yonsei University College of Medicine, Seoul, Republic of Korea; 2 Department of Internal Medicine, Gangnam Severance Hospital, Yonsei University College of Medicine, Seoul, Republic of Korea; 3 Department of Nutrition Care, Gangnam Severance Hospital, Yonsei University Health System, Seoul, Republic of Korea; 4 Cancer Prevention Center, Yonsei Cancer Center, Seoul, Republic of Korea; 5 Department of Radiation Oncology, Yonsei Cancer Center, Yonsei University College of Medicine, Seoul, Republic of Korea

**Keywords:** adiposity, body composition, cachexia, computed tomography, pancreatobiliary cancer, prognostic risk score, sarcopenia

## Abstract

**Background:**

Pancreatobiliary cancers are highly aggressive, with limited prognostic markers in advanced stages. Cancer-associated malnutrition results in sarcopenia and adipose tissue depletion; however, the prognostic implications of comprehensive body composition metrics are unclear.

**Methods:**

We retrospectively analyzed 1,066 metastatic or recurrent pancreatobiliary cancer patients diagnosed between 2006 and 2017. Baseline clinical, laboratory, and nutritional data were collected. Skeletal muscle index (SMI), subcutaneous adipose tissue index (SATI), visceral adipose tissue index (VATI), and muscle attenuation were assessed from computed tomography (CT) images at the third lumbar vertebra (L3). Optimal cut-off values were determined using maximally selected rank statistics. Survival was estimated using the Kaplan–Meier method and compared with the log-rank test. Cox proportional hazards models were used for multivariate analyses.

**Results:**

Sarcopenia was identified in 43% of patients and was associated with older age, male, diabetes mellitus, poor performance status, and unfavorable NRS-2002 score. CT-measured four body composition parameters (SMI, SATI, VATI, muscle attenuation) showed significant prognostic value for overall survival (OS), respectively, with excellent interobserver agreement (ICC range, 0.969–0.995). A composite risk score integrating all the four parameters showed superior prognostic discrimination, with median OS of 11.7, 7.8, and 4.9 months in the low-, moderate-, and high-risk groups (*p* < 0.001). Multivariate analysis confirmed the composite score as an independent predictor of OS and PFS, along with C-reactive protein (CRP) (*p* < 0.001). The composite score retained consistent prognostic value across disease status (recurrent vs. metastatic) and primary tumor subtypes.

**Conclusion:**

Comprehensive CT-based body composition assessment, incorporating sarcopenia, adiposity, and muscle quality indices, improves prognostic stratification in advanced pancreatobiliary cancers. Integrating these body composition metrics with systemic inflammatory markers into routine evaluations may enhance individualized risk stratification and guide treatment decisions.

## 1 Introduction

Pancreatobiliary cancers are among the most aggressive and fatal malignancies worldwide. By 2040, they are projected to become the second and third leading causes of cancer-related deaths (1). In Korea, 17,628 new cases and 13,193 deaths were reported in 2022, making pancreatobiliary cancer the second leading cause of cancer mortality following lung cancer (2). Approximately half of patients present with advanced disease at diagnosis, limiting therapeutic options and highlighting the need for accessible prognostic markers to improve risk stratification and outcome prediction (3, 4). Current prognostic factors in advanced pancreatobiliary cancers are largely confined to primary tumor site, disease extent, performance status (PS), and a few systemic inflammatory markers, including neutrophil-to-lymphocyte ratio (NLR), red blood cell distribution width, and C-reactive protein (CRP) (5–7). However, these do not fully capture the host-related vulnerabilities frequently observed in this population.

Pancreatobiliary cancer patients are usually older and at high risk of malnutrition at diagnosis. As the disease progresses, obstructive complications of the gastric outlet, duodenum, or pancreatic duct frequently occur and require stent placement or bypass surgery (8). Such obstructive complications compound nutritional deterioration, accelerating sarcopenia through cytokine-mediated activation of proteolytic pathways (e.g., ubiquitin–proteasome, autophagy–lysosome systems) and suppression of anabolic signaling, leading to impaired protein synthesis and muscle depletion (9). Concurrently, adipose tissue undergoes catabolic remodeling—characterized by lipolysis, metabolic dysfunction, and the browning of white adipose tissue—resulting in increased energy expenditure and exacerbating negative energy balance (10, 11). These parallel changes in skeletal muscle and adipose tissue reflect the coordinated nature of cancer cachexia, creating a vicious cycle that worsens both nutritional status and treatment outcomes.

Beyond the loss of muscle mass and adipose tissue, the quality of remaining muscle has emerged as a determinant of cancer outcomes. Myosteatosis—defined as ectopic fat infiltration into skeletal muscle—represents an independent marker of muscle quality deterioration that may compound the adverse effects of reduced muscle mass on cancer prognosis (12, 13). Together, sarcopenia, depletion of adipose tissue, and myosteatosis reflect distinct yet interconnected facets of cancer-related body composition alterations, each contributing independently to poor prognosis.

Despite the clinical relevance of these alterations, comprehensive evaluations integrating skeletal muscle quantity, adiposity, and muscle quality remain scarce in advanced pancreatobiliary cancers. Therefore, this study aimed to (1) characterize the clinical and nutritional profiles associated with sarcopenia at diagnosis and (2) evaluate the combined prognostic implications encompassing sarcopenia, adiposity indices, and myosteatosis in patients with advanced pancreatobiliary cancer.

## 2 Materials and methods

### 2.1 Patient population

Patients with pancreatobiliary cancers—originating from the pancreas, bile duct (intrahepatic, extrahepatic, or perihilar), gallbladder, or ampulla of Vater—were included. Patients were diagnosed at Gangnam Severance Hospital, a tertiary cancer center in Seoul, Korea, between September 2006 and July 2017.

Inclusion criteria were: (1) age ≥18 years; (2) histologic confirmation of malignancy; (3) metastatic or recurrent unresectable disease; (4) availability of initial nutritional assessment records, including Nutritional Risk Screening (NRS)-2002 score; 3-month weight loss; and 1-week appetite loss; and (5) access to electronic medical records containing treatment and survival data.

Exclusion criteria included: (1) central nervous system metastasis; (2) localized disease intended for definitive treatment; (3) synchronous advanced malignancy outside the pancreatobiliary system; (4) uncontrolled infection, active bleeding, or severe comorbidities (e.g., advanced liver cirrhosis or chronic kidney disease stage ≥4); and (5) loss to follow-up or transfer to another institution before treatment. The study protocol was approved by the Institutional Review Board of Gangnam Severance Hospital (IRB no. 3-2019-0159); and informed consent was waived because of the retrospective design.

### 2.2 Measurement of body composition metrics

Axial CT images at the third lumbar vertebra (L3) were used to assess body composition. A single slice at the upper border of L3 was analyzed using MIM Vista software (version 6.6.14; MIM Corp., OH, USA). Skeletal muscle, visceral adipose tissue, and subcutaneous adipose tissue were quantified by predefined Hounsfield unit (HU) thresholds: −29 to +150 HU for skeletal muscle, −150 to −50 HU for visceral fat, and −190 to −30 HU for subcutaneous fat. Cross-sectional areas were normalized to height (cm^2^/m^2^) to determine the skeletal muscle index (SMI), visceral adipose tissue index (VATI), and subcutaneous adipose tissue index (SATI). Muscle attenuation was defined as the mean HU of the total skeletal muscle area at L3. Measurements were conducted independently by two investigators (Oh SE and Jeung HC), with all other researchers blinded to the results. Interobserver agreement was assessed using the intraclass correlation coefficient (ICC) with a two-way mixed-effects model (14).

### 2.3 Clinical data collection

Demographic data included age, gender, anthropometry [weight, height, body mass index (BMI)], disease classification—defined as recurrent disease (prior resection followed by documented disease recurrence) or metastatic disease (distant metastases present at initial diagnosis)—primary site and metastasis site, Eastern Cooperative Oncology Group (ECOG)–PS, and presence of diabetes mellitus (DM) or jaundice. Baseline blood tests included white blood cells (WBC), absolute neutrophil count (ANC), hemoglobin (Hb), platelets, CRP, serum protein, albumin, blood urea nitrogen (BUN), aspartate aminotransferase (AST), alanine aminotransferase (ALT), total bilirubin, alkaline phosphatase (ALP), and serum cholesterol. Tumor markers included carcinoembryonic antigen (CEA) and carbohydrate antigen (CA) 19–9. Treatment data, including the type of chemotherapy regimen administered (gemcitabine doublet, other regimens, e.g., gemcitabine monotherapy or fluoropyrimidine-based regimens, or best supportive care only) and time to disease progression was recorded for each patient.

### 2.4 Nutritional risk assessments

The NRS-2002 was assessed at diagnosis by two investigators (Jeung HC and Yoon YE) independently. Per NRS-2002 guidelines, each patient was evaluated for two components (impaired nutritional status and disease severity). They were scored based on whether these components were absent, mild, moderate, or severe, for a total score of 0–6 points, with an adjustment for patients ≥70 years old (15, 16). The final NRS-2002 scores ranged from 0 to 7.

Immuno-nutritional indices, including NLR, advanced lung cancer inflammation index (ALI), nutritional risk index (NRI), prognostic nutritional index (PNI), and systemic immune inflammation index (SII), were calculated from the baseline laboratory data. Definitions for each parameter were described previously (17–19).

### 2.5 Statistical analysis

Hematologic and biochemical parameters were categorized based on their upper normal limits. Each body composition metrics and tumor markers were dichotomized using optimal cut-off values, which were determined by maximally selected rank statistics—an outcome-oriented method identifying the cut-off point most strongly associated with survival (20).

Overall survival (OS) was defined as the time from diagnosis to death from any cause, and progression-free survival (PFS) as the time from diagnosis to disease progression or death. Survival curves were generated using the Kaplan–Meier method and compared by the log-rank test. Significant variables from a univariate analysis were entered into stepwise multivariate models using logistic regression and Cox proportional hazards regression. Hazard ratios (HRs), 95% confidence intervals (CIs), and chi-square statistics were calculated.

To assess potential multicollinearity among the four body composition parameters (SMI, SATI, VATI, and muscle attenuation) included in the multivariable model, variance inflation factors (VIF) were calculated (21).

The analyses were done using PASW Statistics version 18.0 (SPSS Inc., Chicago, IL, USA), SAS version 9.4 (SAS Institute Inc., Cary, NC, USA), and R version 3.2.4 (Institute for Statistics and Mathematics, Vienna, Austria) software. Two-sided *p*-values < 0.05 were considered statistically significant.

## 3 Results

### 3.1 Whole patient demographics and nutritional characteristics

**Figure 1:**
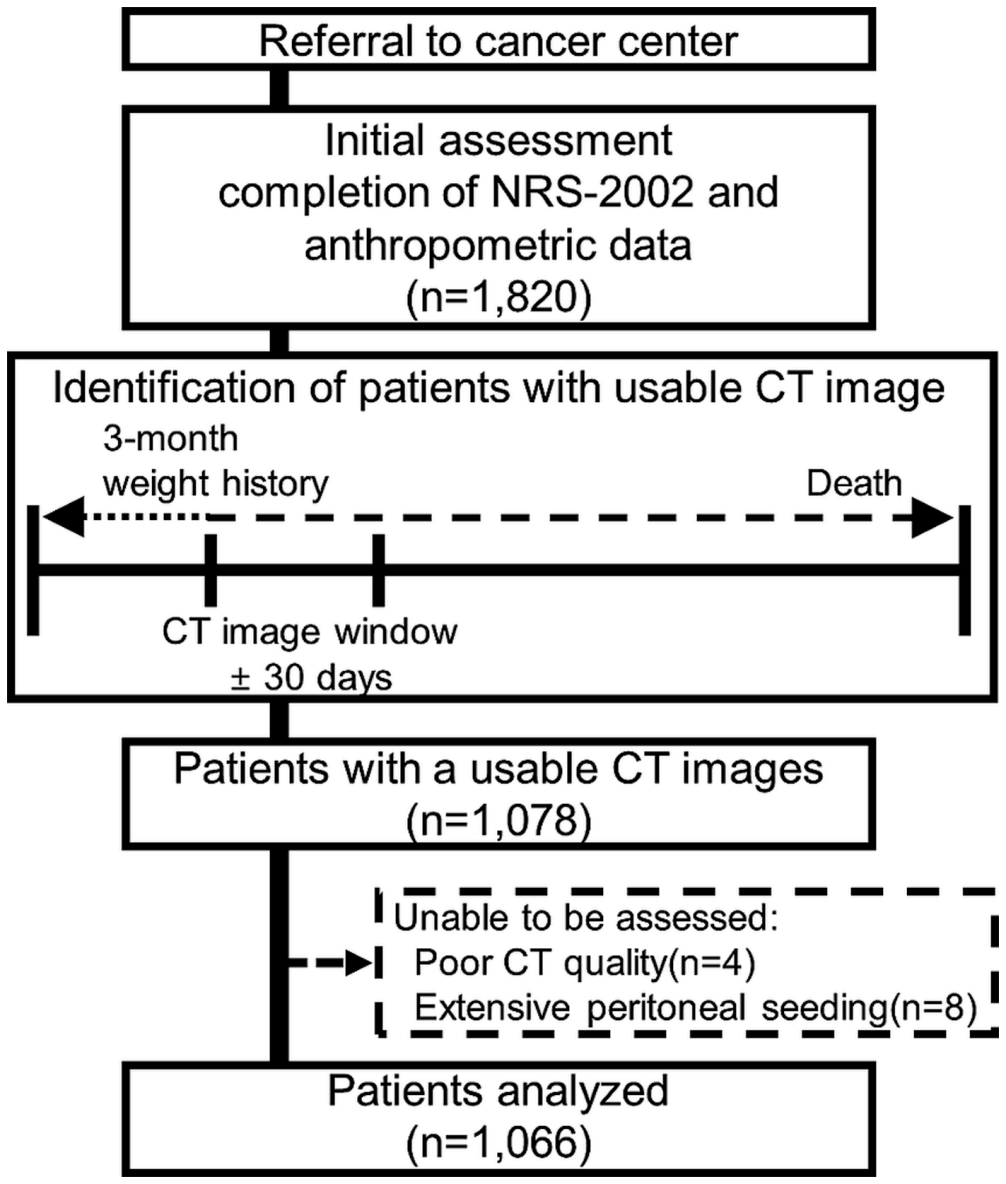
Flow diagram showing study time and patient selection.

Of 1,820 patients initially assessed, 1,078 met eligibility criteria. Twelve patients were subsequently excluded due to inadequate CT image quality precluding reliable segmentation (*n* = 4) or extensive peritoneal seeding with accompanying ascites that rendered body composition measurements unfeasible (*n* = 8), yielding a final cohort of 1,066 patients (Figure 1).

The baseline demographics for the study cohort (*n* = 1,066) are listed in Table 1. The median age was 65 years, 583 patients (55%) were male, and 759 (71%) had metastatic disease at diagnosis. Pancreatic cancer was the most common primary site (44%), followed by bile duct cancer (38%). Liver and peritoneal metastases were present in 42 and 28% of patients, respectively.

**Table 1:** Demographic and clinical parameters for all participants and their comparison between the sarcopenia and non-sarcopenia groups.

Variable	Overall (*N* = 1,066)	Non-Sarcopenia (*N* = 603)	Sarcopenia (*N* = 463)	*p*
Age (*N*, %)				
70 >	674 (63.2)	435 (72.1)	239 (51.6)	<0.001
70 ≤	392 (36.8)	168 (27.9)	224 (48.4)	
Gender (*N*, %)				
Male	583 (54.7)	270 (44.8)	313 (67.6)	<0.001
Female	483 (45.3)	333 (55.2)	150 (32.4)	
Primary site (*N*, %)				
Pancreas	414 (38.8)	209 (34.7)	205 (44.3)	0.013
Bile duct	401 (37.6)	238 (39.5)	163 (35.2)	
Gallbladder	190 (17.8)	119 (19.7)	71 (15.3)	
Periampullary	61 (5.7)	37 (6.1)	24 (5.2)	
Disease status (*N*, %)				
Recurrence	307 (28.8)	200 (33.2)	107 (23.1)	<0.001
Metastasis	759 (71.2)	239 (39.6)	205 (44.3)	
Liver metastasis (*N*, %)				
No	622 (58.3)	364 (60.4)	258 (55.7)	0.128
Yes	444 (41.7)	239 (39.6)	205 (44.3)	
Peritoneal metastasis (*N*, %)				
No	764 (71.7)	440 (73.0)	324 (70.0)	<0.001
Yes	302 (28.3)	163 (27.0)	139 (30.0)	
ECOG-PS (*N*, %)				
0–1	618 (58.0)	392 (65.0)	226 (48.8)	
2–4	448 (42.0)	211 (35.0)	237 (51.2)	
Diabetes mellitus (*N*, %)				
No	651 (61.1)	385 (63.8)	266 (57.5)	0.034
Yes	415 (42.0)	218 (36.2)	197 (42.5)	
Chemotherapy (*N*, %)				
Gemcitabine doublet	646 (60.6)	389 (64.5)	257 (55.5)	<0.001
Other regimens	183 (17.2)	119 (19.7)	64 (13.8)	
Best supportive care	237 (22.2)	95 (15.8)	142 (30.7)	

ECOG-PS, eastern cooperative oncology group-performance status.

Regarding treatment, 646 patients (61%) received gemcitabine doublet, 183 (17%) with other regimens, and 237 (22%) received best supportive care only, in consideration of performance status or patient preference. The proportion of patients receiving no active treatment was significantly higher in the sarcopenic group (31%) than in the non-sarcopenic group (16%).

Nutritional and laboratory information at diagnosis is listed in Table 2. The mean BMI for the whole cohort was 22.6 kg/m^2^, and 95 patients (9%) were classified as underweight (BMI < 18.5 kg/m^2^), whereas 231 (22%) were overweight or obese (BMI ≥ 25 kg/m^2^). The prevalence of overweight or obesity was similar between men (22%) and women (21%) (*p* = 0.862). At the initial nutritional assessment, 416 patients (39%) reported involuntary weight loss of >5% within 3 months, and 358 (34%) reported persistent inappetence lasting more than 1 week.

**Table 2:** Nutritional and laboratory parameters for all patients and their comparison between the sarcopenia and non-sarcopenia groups.

Variable	Overall *N* = 1,066	Non-Sarcopenia *N* = 603	Sarcopenia *N* = 463	*p*
Body composition data (M, Min-Max)				
Height (cm)	161.2 (135.0–185.0)	159.7 (135.0–185.0)	163.2 (140.0–185.0)	< 0.001
Weight (Kg)	59.1 (31.0–97.1)	60.9 (35.0–97.1)	56.7 (31.0–84.8)	< 0.001
BMI (Kg/M^2^) (M, Min-Max)	22.6 (12.1–35.6)	23.8 (15.8–35.6)	21.2 (12.1–29.9)	< 0.001
18.5 > (*N*, %)	95 (8.9)	19 (3.2)	76 (16.4)	< 0.001
18.5 ~ 25.0 (*N*, %)	740 (69.4)	395 (65.5)	345 (74.5)	
25.0 < (*N*, %)	231 (21.7)	189 (31.3)	42 (9.1)	
3-month weight loss (*N*, %)				
No	650 (61.0)	408 (67.7)	242 (52.3)	< 0.001
Yes	416 (39.0)	195 (32.3)	221 (47.7)	
1-week inappetence (*N*, %)				
No	708 (66.4)	439 (72.8)	269 (58.1)	< 0.001
Yes	358 (33.6)	164 (27.2)	194 (41.9)	
NRS-2002 score (*N*, %)				
1–2	573 (53.8)	397 (65.8)	176 (38.0)	< 0.001
3–6	493 (46.2)	206 (34.2)	287 (62.0)	
Complete blood count (M, Min-Max)				
WBC (10^3^/μL)	7.82 (1.99–44.24)	7.48 (2.54–29.93)	8.28 (1.99–44.24)	< 0.001
Hemoglobin (g/dL)	12.6 (4.6–156.9)	12.9 (5.1–156.9)	12.1 (4.6–17.4)	0.031
Platelet (10^3^/μL)	259 (54–827)	260 (63–690)	258 (54–827)	0.690
ANC (10^3^/μL)	5.42 (0.78–41.20)	5.04 (0.96–25.82)	5.91 (0.78–41.20)	< 0.001
Lymphocyte (10^3^/μL)	1.59 (0.14–5.28)	1.66 (0.14–4.60)	1.51 (0.22–5.28)	< 0.001
Serum biochemistry (M, Min-Max)				
Total Protein (g/dL)	6.9 (4.2–9.0)	6.9 (4.4–8.8)	6.8 (4.2–9.0)	< 0.001
Albumin (g/dL)	3.9 (1.7–5.7)	4.0 (1.7–5.7)	3.8 (2.0–5.4)	< 0.001
ALP (IU/L)	242 (30–2,212)	230 (30–1898)	258 (34–2,212)	0.072
AST(GOT) (IU/L)	66 (10–905)	66 (10–621)	67 (10–905)	0.859
ALT(GPT) (IU/L)	69 (4–933)	68 (4–669)	71 (5–933)	0.632
CRP (mg/L)	31.5 (0.1–456.2)	25.9 (0.1–275.0)	38.6 (0.1–456.2)	< 0.001
CEA (ng/mL)	72.0 (0.1–8281.0)	69.7 (0.4–8281.0)	75.0 (0.1–8168.6)	0.681
CA19-9 (U/mL)	2586.3 (0.6–25160.0)	2204.5 (0.6–20850.0)	3089.2 (0.6–25160.0)	0.010
BUN (mg/dL)	15.8 (3.0–82.6)	14.7 (3.0–71.2)	17.2 (4.2–82.6)	< 0.001
Bilirubin (mg/dL)	3.0 (0.1–38.5)	2.6 (0.1–29.2)	3.6 (0.2–38.5)	0.008
Nutritional index (M, Min-Max)				
NLR	4.4 (0.3–117.7)	3.8 (0.5–33.0)	5.3 (0.3–117.7)	< 0.001
PNI	47.2 (19.5–66.8)	48.3 (19.5–66.8)	45.7 (23.1–66.4)	< 0.001
NRI	103.4 (64.6–139.5)	107.2 (73.6–139.5)	98.5 (64.6–125.6)	< 0.001
ALI	36.4 (0.6–220.7)	41.2 (1.9–220.7)	30.2 (0.6–180.4)	< 0.001
SII	1165.7 (105.4–23425.1)	1016.5 (106.8–11929.8)	1359.9 (105.4–23425.1)	< 0.001

BMI, body mass index; NRS-2002, nutritional risk screening-2002; WBC, white blood cell; ANC, absolute neutrophil count; ALP, alkaline phosphatase; AST, aspartate aminotransferase; ALT, alanine transaminase; CRP, C-reactive protein; CEA, carcinoembryonic antigen; CA19-9, carbohydrate antigen 19–9; BUN, blood urea nitrogen; NLR, neutrophil-to-lymphocyte ratio; PNI, prognostic nutritional index; NRI, nutritional risk index; ALI, advanced lung cancer inflammation index; SII, systemic immune inflammation index.

### 3.2 Demographic and clinical characteristics according to sarcopenia status

ICC value for CT-based body composition measurements was 0.987 (95% CI, 0.974–0.995) for SMI, 0.995 (0.990–0.998) for SATI, 0.994 (0.988–0.998) for VATI, and 0.969 (0.936–0.987) for muscle attenuation.

Cross-sectional CT imaging at L3 was used for body composition analysis. The mean SMI value was 44.86 cm^2^/m^2^ in men and 36.10 cm^2^/m^2^ in women (Table 3). A moderate positive correlation between SMI and BMI was observed in men (r^2^ = 0.25, *p* < 0.001) and women (r^2^ = 0.21, *p* < 0.001) (Supplementary Figure 1). Using maximally selected rank statistics, cut-off values for sarcopenia were defined as SMI < 45.2 cm^2^/m^2^ in men and SMI < 32.9 cm^2^/m^2^ in women, thus resulting in the classification of 463 patients (43%) with sarcopenia (68% men and 32% women, *p* < 0.001).

**Table 3:** CT-obtained body composition metrics for all patients and their comparison between the sarcopenia and non-sarcopenia groups.

Variable	Overall			Non-sarcopenia			Sarcopenia			*p*
	Total *N* = 1,066	Male *N* = 583	Female *N* = 483	Total *N* = 603	Male *N* = 270	Female *N* = 333	Total *N* = 463	Male *N* = 313	Female *N* = 150	
SMI (cm^2^/m^2^) (Mean, Min-Max)	40.89 (15.67–74.53)	44.86 (16.34–74.53)	36.10 (15.67–63.31)	44.99 (33.02–74.53)	51.97 (45.33–74.53)	39.32 (33.02–63.31)	35.55 (15.67–45.24)	38.72 (16.34–45.24)	28.95 (15.67–32.92)	< 0.001
SATI (cm^2^/m^2^) (Mean, Min-Max)	40.38 (0.13–158.24)	30.81 (0.13–151.93)	51.86 (0.88–158.24)	47.75 (3.02–158.24)	36.43 (3.02–151.93)	56.85 (3.40–158.24)	30.67 (0.13–128.97)	25.93 (0.13–100.61)	40.60 (0.88–128.97)	< 0.001
VATI (cm^2^/m^2^) (Mean, Min-Max)	33.50 (0.50–232.31)	33.52 (0.50–113.02)	33.49 (0.50–232.31)	36.86 (1.54–113.44)	39.36 (2.83–113.02)	34.86 (1.54–113.44)	29.08 (0.50–232.31)	28.45 (0.50–99.93)	30.40 (0.50–232.31)	< 0.001
Muscle Attenuation (HU) (Mean, Min-Max)	33.78 (1.24–86.86)	36.94 (14.28–86.86)	29.97 (1.24–59.91)	34.39 (12.80–59.91)	38.26 (17.48–59.33)	31.26 (12.80–59.91)	32.98 (1.24–86.86)	35.79 (14.28–86.86)	27.06 (1.24–54.84)	0.015

SMI, skeletal muscle index; SATI, subcutaneous adipose tissue index; VATI, visceral adipose tissue index; HU, hounsfield unit.

Demographic characteristics were further analyzed between the population with and without sarcopenia (Table 1). Sarcopenia was associated with males, older age (≥70 years, *p* < 0.001), poor performance (ECOG-PS ≥ 2, *p* < 0.001), and the presence of DM (*p* = 0.013). In contrast, sarcopenia was not significantly affected by the presence of metastases or primary sites (pancreas vs. biliary tract). Regarding the laboratory findings, sarcopenic patients exhibited higher WBC, ANC, and CRP levels (*p* < 0.001, respectively), whereas lymphocyte and platelet counts were lower. Moreover, mean serum albumin and protein levels were distinctively reduced in the sarcopenia group (*p* < 0.001).

### 3.3 Association between sarcopenia and nutritional parameters

Table 2 lists the nutritional profiles of the population according to sarcopenia. The sarcopenic population had a lower mean BMI, a markedly higher prevalence of being underweight, and was more likely to report involuntary weight loss over 3 months and inappetence longer than 1 week at the initial nutritional consultation. When high nutritional risk was defined as an NRS-2002 score ≥3, 62% of the sarcopenic population was classified as high risk (*p* = 0.001). Logistic regression identified older age (>65 years; odds ratio (OR), 2.046; *p* < 0.001), high NRS-2002 score (≥3; OR, 2.932; *p* < 0.001), and male gender (OR, 4.354; *p* = 0.001) as the strongest predictors of sarcopenia. Additional associations were observed for DM, anemia, and poor PS (Supplementary Table 1).

Next, we examined the relationship between sarcopenia and immunonutritional indices. Of the indices evaluated (NLR, PNI, NRI, ALI, and SII), patients with sarcopenia showed significant differences compared with their counterparts, suggesting that sarcopenia at the time of diagnosis reflects an already compromised systemic immune response (Supplementary Figure 2).

### 3.4 Gender-specific differences in adiposity and muscle attenuation

VATI, SATI, and muscle attenuation were evaluated concurrently with SMI. Their mean values and gender-specific differences are listed in Table 3. No significant differences in VATI were observed between the male and female groups, whereas SATI was higher in women (51.86 vs. 30.75 cm^2^/m^2^, *p* < 0.001), and muscle attenuation was higher in men (36.94 vs. 29.97 HU, *p* < 0.001). Compared with the non-sarcopenic group, those with sarcopenia exhibited significantly lower mean values of VATI (29.08 vs. 36.86 cm^2^/m^2^, *p* < 0.001), SATI (30.07 vs. 47.75 cm^2^/m^2^, *p* < 0.001), and muscle attenuation (32.98 vs. 34.39 HU, *p* = 0.015). A scatter plot revealed correlations between SMI and these adiposity indices (Supplementary Figure 3). A modest correlation was observed between SMI and SATI (r^2^ = 0.0187, *p* = 0.019), whereas moderate correlations were shown between SMI and VATI (r^2^ = 0.0662, *p* < 0.001) and between SMI and muscle attenuation (r^2^ = 0.1024, *p* < 0.001).

### 3.5 Survival outcomes based on the composite anthropometric risk scoring

The median follow-up duration for the entire cohort was 9.2 months (range, 0.2–135 months), and the median OS was 9.3 months (95% CI, 8.6–10.0 months). When stratified by the BMI categories, overweight or obese patients exhibited the longest OS of 11.6 months (95% CI, 9.9–13.3), compared with 9.2 months (95% CI, 8.5–9.9) for those with normal BMI and 5.7 months (95% CI, 3.8–7.6) for underweight patients (*p* < 0.001) (Supplementary Figure 4).

**Figure 2:**
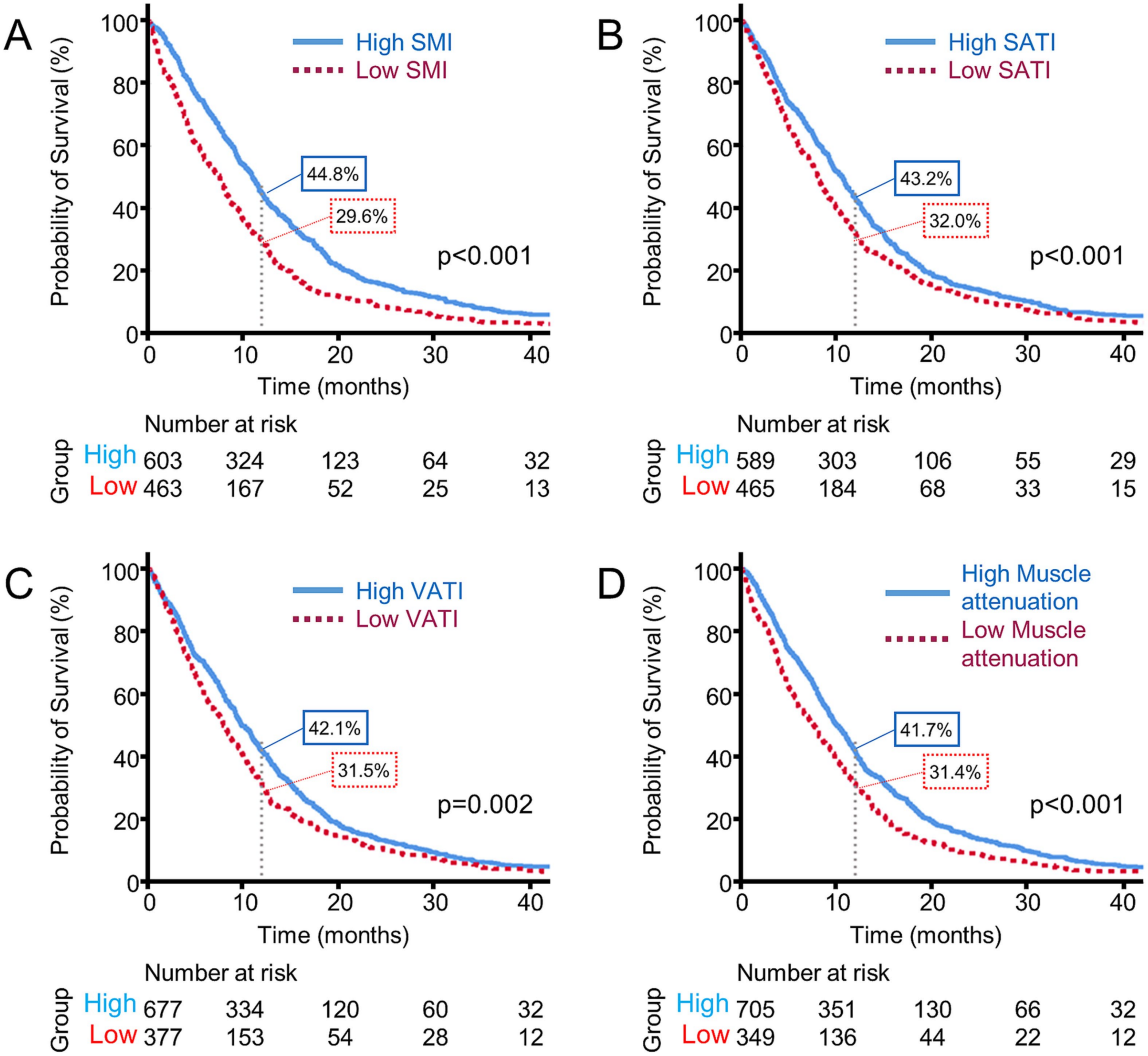
Comparison of survival curves based on CT-determined parameters: **(A)** SMI; **(B)** SATI; **(C)** VATI; **(D)** Muscle attenuation.

Then, survival analysis was done based on SMI, which revealed a median OS of 8.3 months (95% CI, 7.7–8.9) in the sarcopenia group vs. 12.8 months (95% CI, 11.6–14.0) in the non-sarcopenia group (*p* < 0.001). The 1-year OS rate was 29.6% in the sarcopenia group compared with 44.8% in the non-sarcopenia group (*p* = 0.020; Figure 2A).

The optimal cut-off values for SATI (32.50 cm^2^/m^2^), VATI (22.00 cm^2^/m^2^), and muscle attenuation (29.99 HU) were derived using maximally selected rank statistics. Patients with low SATI exhibited an inferior OS (8.2 months; 95% CI, 7.4–9.0 vs. 10.7 months; 95% CI, 9.9–11.5; *p* = 0.001). Alongside, low VATI was associated with a shorter OS (8.0 months; 95% CI, 7.1–8.9 vs. 10.2 months; 95% CI, 9.4–11.0; *p* = 0.002), and low muscle attenuation (7.6 months; 95% CI, 6.3–8.9 vs. 10.1 months; 95% CI, 9.3–10.9; *p* < 0.001; Figures 2B–D). Taken together, each body composition parameter (SMI, SATI, VATI, and muscle attenuation) independently contributed to OS.

Next, we hypothesized that the integration of the body composition parameters at diagnosis provides better prognostic stratification compared with individual metrics. To assess potential multicollinearity among the four body composition parameters included in the multivariable model, VIF values were calculated. VIF values were 1.28 for SMI, 1.63 for SATI, 1.70 for VATI, and 1.48 for muscle attenuation, confirming that multicollinearity was not a significant concern. After fitting a multivariable Cox proportional hazards model incorporating four key continuous variables, an integrated risk score was calculated for each individual patient using the regression coefficients of the model as weights; SMI × (−0.0164) + SATI × (−0.0056) + VATI × (−0.0014) + muscle attenuation × (−0.0147) Based on the derived risk scores, two optimal cut-off points (−1.274 and −0.105) were identified using a grid search approach that maximized the log-rank test statistic, and patients were accordingly stratified into three risk groups (low-risk, moderate-risk, and high-risk). To assess the potential risk of overfitting associated with data-driven cut-off point selection using the same dataset, a bootstrap procedure was performed. The distribution and confidence intervals of the cut-off points obtained across bootstrap iterations were examined to evaluate the stability and reproducibility of the cut-off point selection.

**Figure 3:**
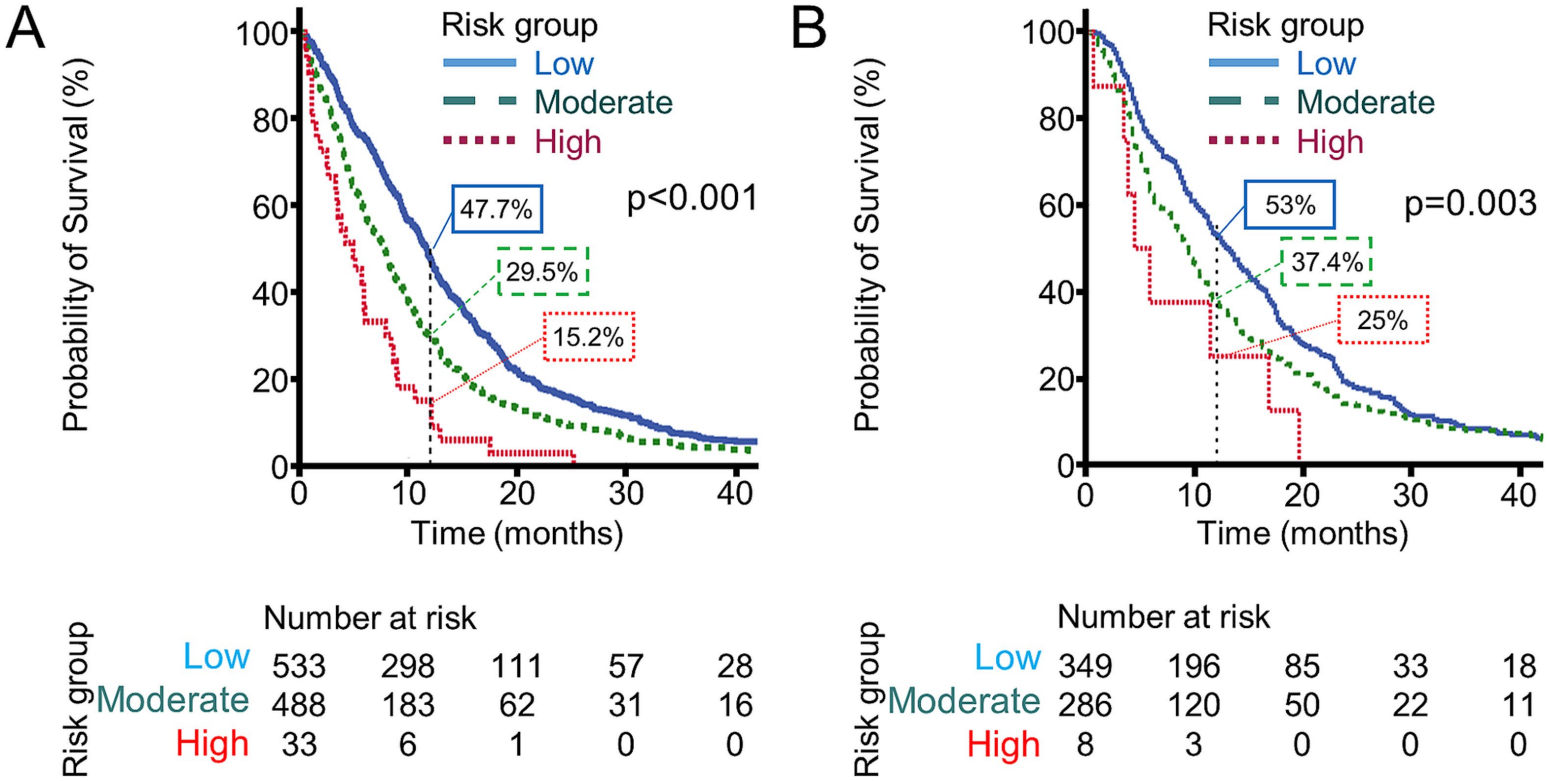
Survival curves based on the combination of CT-determined parameters: **(A)** overall survival; **(B)** progression-free survival.

There was a significant difference in OS among the groups, with a median OS of 11.7 months (95% CI, 10.9–12.5) in the low-risk group, 7.8 months (95% CI, 7.0–8.6) in the moderate-risk group, and only 4.9 months (95% CI, 2.4–7.4 months) in the high-risk group (*p* < 0.001; Figure 3A). These results indicate that composite anthropometric risk scoring provides superior prognostic discrimination compared with individual body composition indices or BMI alone.

We further determined whether this composite risk score could be applied to PFS in patients treated with gemcitabine-doublet chemotherapy (*n* = 643). Median PFS was 6.5 months (95% CI, 5.7–7.3) in the low-risk group, 4.7 months (95% CI, 4.1–5.3) in the moderate-risk group, and only 2.2 months (95% CI, 0.8–3.6) in the high-risk group (*p* = 0.003; Figure 3B). Collectively, these results indicate that this composite risk scoring incorporating SMI, SATI, VATI, and muscle attenuation offers prognostic value, with higher risk categories associated with shorter OS and PFS.

To assess the robustness of the composite risk score, subgroup analysis was done by disease status (recurrent vs. metastatic) and primary tumor site (pancreas vs. biliary tract). Among patients with metastatic disease, the composite risk score remained significantly associated with OS, with median OS of 10.2 months in the low-risk group, 6.1 months in the moderate-risk group, and 4.2 months in the high-risk group (*p* < 0.001). A similar pattern was also observed in patients with recurrent disease, with median OS of 14.3, 10.9, and 4.9 months in the low-, moderate-, and high-risk groups, respectively (*p* < 0.001). When stratified by primary tumor site, the prognostic value was consistent both in pancreatic cancer (*p* < 0.001) and biliary tract cancers (*p* < 0.001) (Supplementary Figure 5).

### 3.6 Multivariate analysis of independent prognostic factors

Finally, multivariate analysis indicated that this composite risk score was independently associated with OS. Compared with the low-risk group, patients in the moderate-risk group exhibited an HR of 1.359 (95% CI, 1.191–1.551; *p* < 0.001) and those in the high-risk group had an HR of 1.995 (95% CI, 1.392–2.859; *p* = 0.001). Among the additional independent prognostic factors identified, elevated CRP (>6 mg/L; HR, 1.483; 95% CI, 1.281–1.717; *p* < 0.001) was a particularly strong predictor of OS, underscoring the prognostic importance of systemic inflammation alongside with body composition. Elevated CEA (>9 ng/mL; HR, 1.524; 95% CI, 1.343–1.752; *p* < 0.001), and SII (HR, 1.574; 95% CI, 1.374–1.832; *p* < 0.001) were also independently associated with OS (Table 4).

**Table 4:** Univariate and multivariate analyses for overall survival.

Variable	Univariate analysis			Multivariate analysis		
	HR	95% CI	*p*	HR	95% CI	*p*
BMI (Kg/M^2^)						
<18.5/18.5–25.0	1.435	1.159–1.777	0.001			
>25.0/18.5–25.0	0.857	0.738–0.996	0.044			
Combining risk group						
Moderate/Low	1.426	1.258–1.618	<0.001	1.359	1.191–1.551	<0.001
High/Low	2.713	1.903–3.868		1.995	1.392–2.859	<0.001
WBC (10^3^/μL)						
< 4,000/ ≥4,000	0.936	0.728–1.203	0.606			
Hemoglobin (g/dL)						
<13/≥13	1.232	1.085–1.399	0.001			
SII						
≥953.92/<953.92	2.062	1.816–2.341	<0.001	1.574	1.357–1.827	<0.001
Total Protein (g/dL)						
<6.9/≥6.9	1.267	1.121–1.431	<0.001			
CRP (mg/L)						
>6.0/≤6.0	1.834	1.611–2.089	<0.001	1.483	1.281–1.717	<0.001
CEA (ng/mL)						
>5.0/≤5.0	1.673	1.476–1.896	<0.001	1.534	1.343–1.752	<0.001
CA19-9 (U/mL)						
>37.0/≤37.0	1.318	1.148–1.512	<0.001			

BMI, body mass index; WBC, white blood cell; SII systemic immune inflammation index; CRP, C-reactive protein; CEA, carcinoembryonic antigen; CA19-9, carbohydrate antigen 19–9.

For PFS, composite risk score also demonstrated independent value, with HRs of 1.276 (95% CI, 1.072–1.519; *p* = 0.006) in the moderate-risk group and 2.052 (95% CI, 1.014–4.154; *p* = 0.046) in the high-risk group. CRP elevation and lymphopenia were also considered adverse predictors of PFS. Taken together, these results highlight the prognostic significance of integrating sarcopenia and body adiposity parameters and their combined impact on survival in patients with advanced pancreatobiliary cancer (Supplementary Table 2).

## 4 Discussion

This large retrospective study of 1,066 patients with advanced pancreatobiliary cancer demonstrates that CT-based body composition assessment at diagnosis—encompassing SMI, SATI, VATI, and muscle attenuation—provides meaningful and independent prognostic stratification. The composite risk score identified three groups with substantially different survival outcomes with median OS of 11.7, 7.8, and 4.9 months in the low-, moderate-, and high-risk groups (*p* < 0.001), and this prognostic value was maintained in multivariate analysis alongside established inflammatory biomarkers including CRP and SII. Subgroup analyses confirmed consistent composite score performance across disease status and primary tumor subtype, supporting its applicability across the pancreatobiliary cancer spectrum.

Sarcopenia is part of the systemic cytokine-mediated inflammatory response. It results in a pro-catabolic state, in which proteolysis, gluconeogenesis, and eventual skeletal muscle depletion occur (9–11). Despite the widespread perception that pancreatobiliary cancer results in significant nutritional decline, the true prevalence of sarcopenia remains unclear. In the present study, we demonstrated that 43% of the patients were already sarcopenic at diagnosis, which underscores its clinical relevance. Nevertheless, interpreting this figure requires caution because of the heterogeneity of the cut-off criteria used to define sarcopenia. CT imaging at the L3 vertebra level has been used in many studies; however, each researcher has applied different criteria for the truncation point. In particular, the criteria established by the international guidelines of an SMI < 55 cm^2^/m^2^ for males and <39 cm^2^/m^2^ for females are primarily based on European and American patients (22). An Asian-specific consensus has proposed alternative criteria that account for the smaller muscle mass typically observed in Asian populations (23), and Japan Society of Hepatology guidelines defined sarcopenia as an SMI < 42 cm^2^/m^2^ for men and <38 cm^2^/m^2^ for women (24). In the present study, we adopted maximally selected rank statistics to derive cut-off values for body composition indices (SMI < 45.2 cm^2^/m^2^ in men and <32.9 cm^2^/m^2^ in women). This outcome-oriented, data-driven method identifies thresholds that are strongly associated with survival, thereby improving prognostic discrimination compared with arbitrary or median-based splits. We did consider published Asian-specific cut-offs; however, given the advanced cancer context and the survival-based optimization goal of this study, we prioritized outcome-oriented thresholds. Nonetheless, it has inherent risks of overfitting and the loss of information from dichotomization. Its generalizability also remains limited without validation in independent cohorts. These limitations may be overcome through validation in independent cohorts or cross-cancer studies.

Beyond sarcopenia, the present study highlights the interplay between malnutrition, systemic inflammation, and survival in advanced pancreatobiliary cancer. Among the inflammatory biomarkers evaluated, elevated CRP (>6 mg/L) emerged as the second strongest independent predictor of OS (HR, 1.483; 95% CI, 1.281–1.717; *p* < 0.001). This finding is consistent with the well-established role of CRP as a marker of systemic inflammatory burden in cancer cachexia, where pro-inflammatory cytokines—including interleukin (IL)-6 and tumor necrosis factor (TNF)-*α*—drive concurrent muscle wasting and adipose tissue remodeling (10). The SII (HR, 1.574; *p* < 0.001), which integrates neutrophil, platelet, and lymphocyte counts, similarly captures this inflammatory milieu. These findings suggest that body composition metrics and inflammatory biomarkers provide complementary and additive prognostic information, and their combined assessment in routine clinical practice may substantially improve risk prediction.

Our observation that higher SATI and VATI values are correlated with improved survival is consistent with the ‘obesity paradox,’ in which overweight or obese patients exhibit longer survival compared with underweight or even normal BMI patients—a phenomenon described across multiple cancer types including lung cancer, renal cell carcinoma, and melanoma (25). Visceral fat shows increased lipolytic activity and releases pro-inflammatory cytokines, including IL-6 and TNF-*α*, with direct hepatic exposure through portal circulation (26–28). Conversely, subcutaneous fat is a key source of leptin, serves as an energy reservoir that may buffer cancer-induced hypercatabolism, and contributes to improved insulin sensitivity (29, 30). These divergent roles reinforce the need for the individualized assessment of fat deposits in prognostic models.

Muscle attenuation, which indicates myosteatosis or fat infiltration within muscle tissue, is a marker of decreased muscle quality. Myosteatosis is an important independent predictor of reduced survival rates in various cancers, such as colorectal, lung, and gastric cancers. Myosteatosis has emerged as an increasingly recognized prognostic marker across multiple cancer types, and has been shown to predict not only reduced survival but also increased treatment toxicity (13). Recent studies have proposed integrating serum albumin with myosteatosis into composite indices—such as the albumin-myosteatosis gauge—which demonstrated significant prognostic value in gastrointestinal cancers (17). This concept of combining nutritional and body composition parameters closely aligns with the composite risk score approach employed in the present study. In the present study, low muscle attenuation also correlated with poor outcomes, supporting its incorporation along with muscle mass in prognostic frameworks. The relationship between adiposity and muscle attenuation may reflect altered lipid trafficking, increased fatty acid availability, and ectopic fat deposition within muscle (31, 32). Future studies should explore whether targeted interventions—such as exercise training or nutritional supplementation—can reverse myosteatosis and improve patient outcomes.

A notable strength of this study is the introduction of an innovative anthropometric risk scoring model that incorporates sarcopenia, SATI, VATI, and muscle attenuation. The reliability of CT-derived body composition measurements in this study is supported by high ICC values, consistent with prior studies validating CT-based indices against reference methods such as dual-energy X-ray absorptiometry and bioelectrical impedance analysis (33–35). This composite risk stratification model has demonstrated superior power in classifying patients into low-, moderate-, and high-risk categories, compared with single indices or BMI. For example, this scoring system may be integrated into a more sophisticated treatment decision-making framework: low-risk patients may benefit from more aggressive therapeutic strategies such as doublet or triplet chemotherapy or enrollment in clinical trials with novel agents, whereas high-risk patients are unlikely to tolerate intensive regimens and may be better served by early palliative and supportive care planning. The consistent prognostic performance across disease status and primary tumor subtypes further supports the broad applicability of this scoring system.

The present study has several limitations. First, the retrospective, single-center design limits its generalizability, and the derived cutoffs require prospective validation. Variations in imaging protocols and the acquisition of laboratory data also represent potential sources of bias. Second, combining pancreatic, bile duct, gallbladder, and periampullary cancers into a single cohort introduces biological heterogeneity. Although our subgroup analyses demonstrated consistent prognostic performance of the composite score across tumor subtypes and disease status groups, these analyses were limited by small high-risk subgroup sizes, and caution is warranted in generalizing results to individual tumor types. Third, treatment allocation was not randomized, and 22% of patients received best supportive care only, predominantly reflecting poor performance status; sensitivity analyses including treatment type as a covariate confirmed the robustness of the composite score (moderate-risk group HR, 1.269, 95% CI, 1.109–1.451, *p* < 0.001; high-risk group HR, 1.522, 95% CI, 1.054–2.196, *p* = 0.025), although residual confounding cannot be excluded. Another limitation is that PFS was only evaluated in patients administered gemcitabine-doublet chemotherapy. Considering the enrollment period of this study, the standard regimens for pancreatobiliary cancer at the time were gemcitabine monotherapy or gemcitabine-based doublet combinations. Therefore, our results may not fully reflect the updated survival spectrum achieved with contemporary treatments, such as FOLFIRINOX (oxaliplatin, irinotecan, fluorouracil, and leucovorin) or immune checkpoint inhibitors. Whether the cut-off points established in the present study remain applicable to more recently treated patient populations warrants further study.

Future studies should focus on prospective, multicenter validation of the anthropometric risk model, integration of functional capacity assessments such as handgrip strength and physical activity monitoring, mechanistic studies to determine the association between inflammation, metabolism, and body composition, and clinical trials evaluating multimodal interventions—including nutritional supplementation, anti-inflammatory therapies, and exercise training—to translate these findings into improved patient care.

## 5 Conclusion

Our study provides strong evidence that comprehensive CT-based body composition assessment at diagnosis, which incorporates sarcopenia, SATI, VATI, and muscle attenuation, offers superior prognostic value for advanced pancreatobiliary cancers. The composite risk score enhances prognostic accuracy, demonstrates consistent performance across disease status and primary tumor subtype subgroups, and supports individualized clinical decision-making. The independent prognostic contribution of systemic inflammatory markers, particularly CRP, underscores the need for multidimensional approaches that combine body composition assessment with inflammatory biomarkers. Future prospective multicenter validation studies and clinical trials evaluating multimodal interventions—including nutritional support, anti-inflammatory therapies, and exercise training—are warranted to translate these findings into improved patient care.

## Data Availability

The raw data supporting the conclusions of this article will be made available by the authors, without undue reservation.
