# Supplementary material for: Integrated CT-based assessment of muscle and adiposity for risk stratification in advanced pancreatobiliary cancer
Source: Front Nutr. 2026 May 14;13:1792814. doi: 10.3389/fnut.2026.1792814 (PMC13219963; doi:10.3389/fnut.2026.1792814)
Supplement: Supplementary file 1 [file Table_1.docx]

Supplementary Material

**
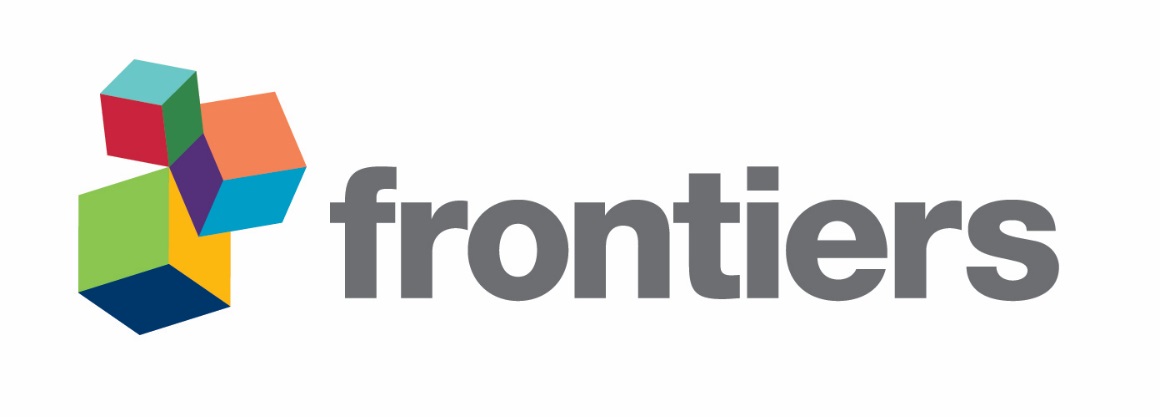
**

Supplementary Figure 1. Correlation between skeletal muscle index (SMI) and body mass index (BMI) according to sex.

(A) Male, (B) Female.


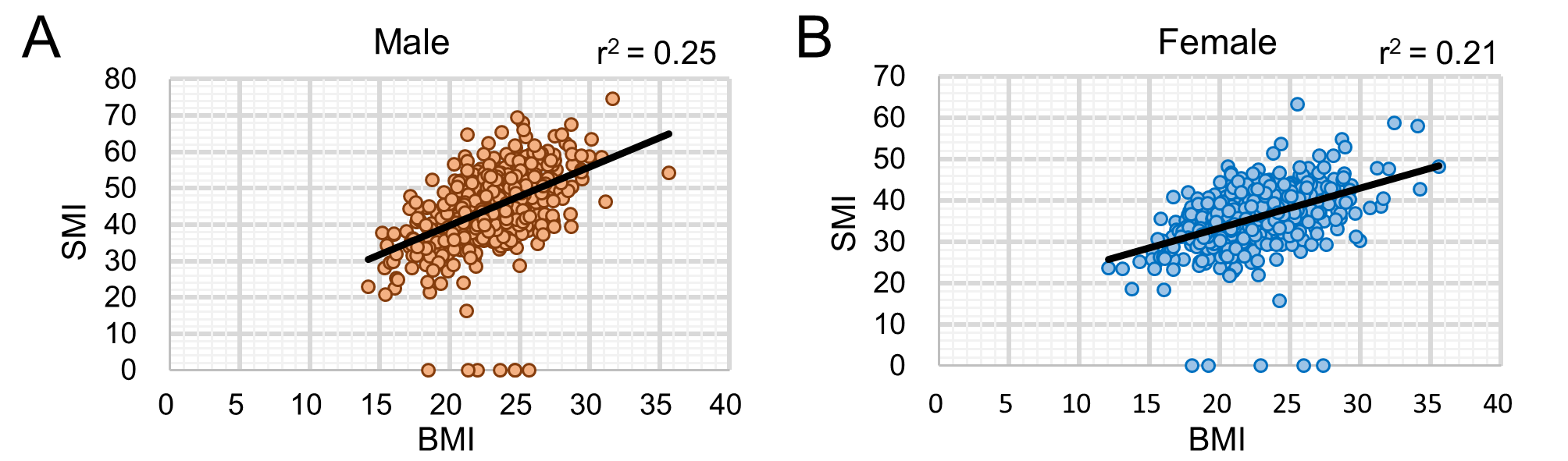


Supplementary Figure 2. Relationship between skeletal muscle index (SMI) and several immunonutritional indices.

(A) PNI: prognostic nutritional index, (B) NRI: nutritional risk index, (C) ALI: Advanced Lung Cancer Inflammation Index, and (D) SII: Systemic Immune-Inflammation Index.


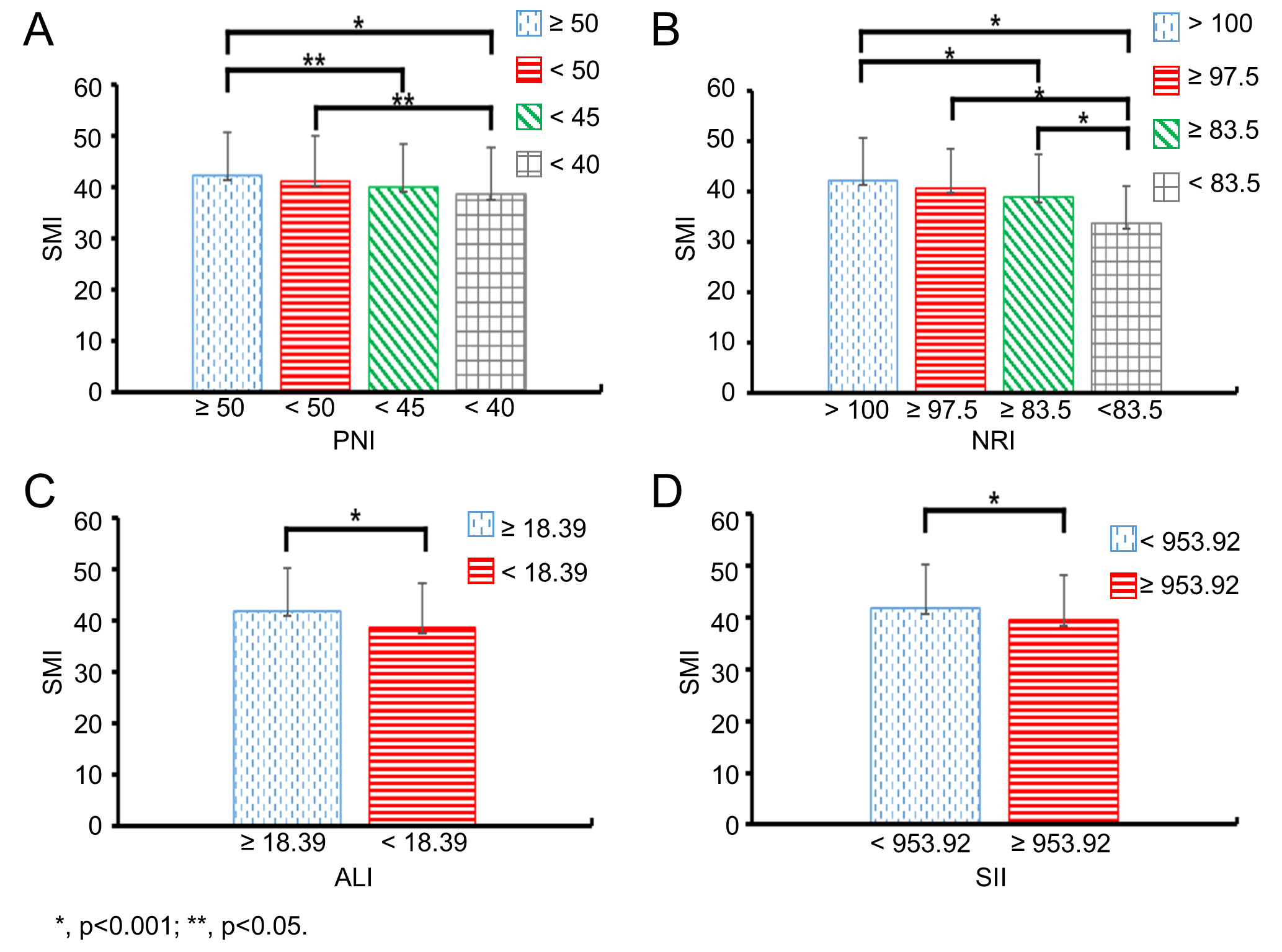


Supplementary Figure 3. Scatter plot between (A) Skeletal muscle index (SMI) and subcutaneous adipose tissue index (SATI); (B) SMI and visceral adipose tissue index (VATI); (C) SMI and muscle attenuation; and (D) SATI and VATI.


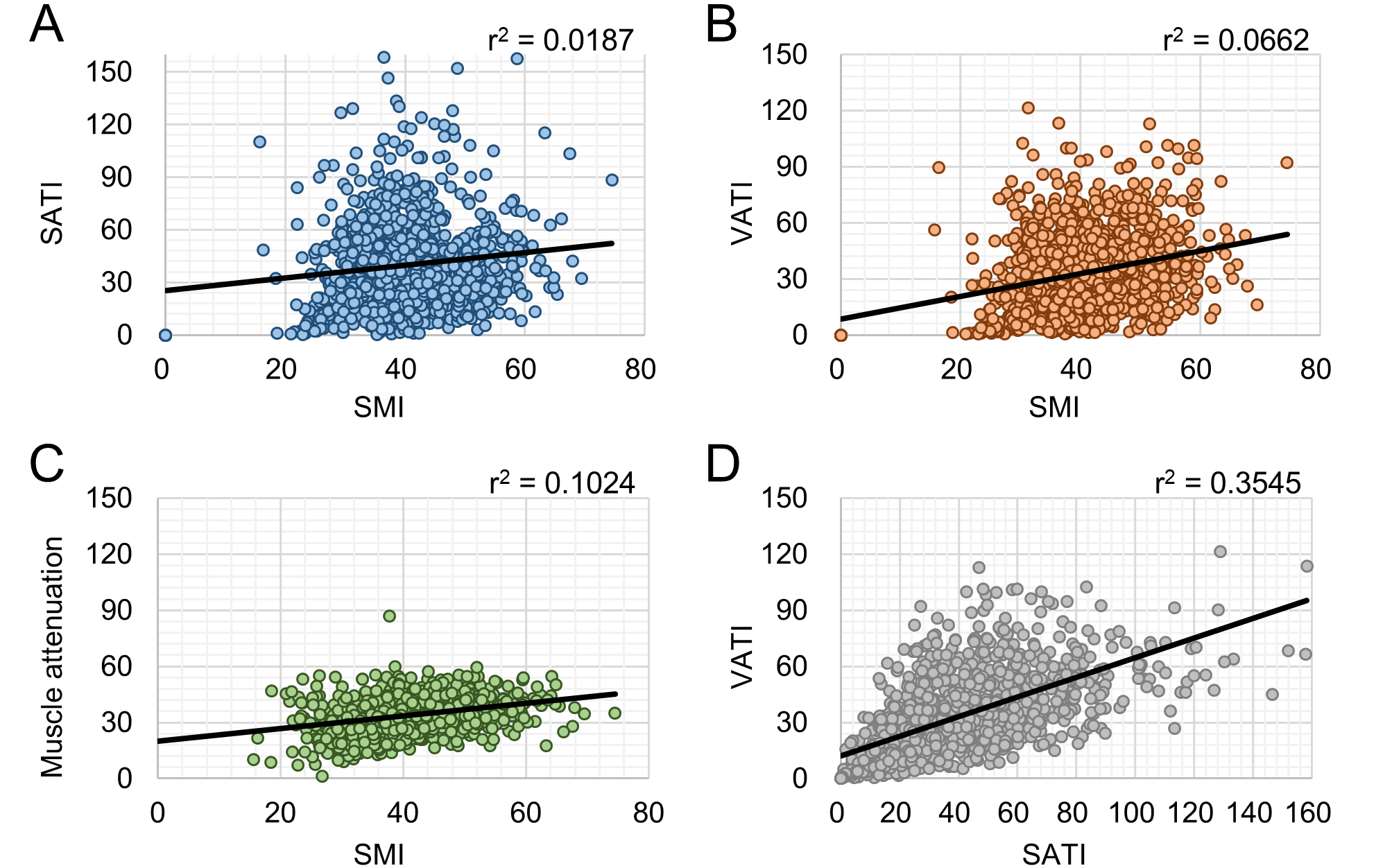


Supplementary Figure 4. Overall survival according to body mass index (BMI).


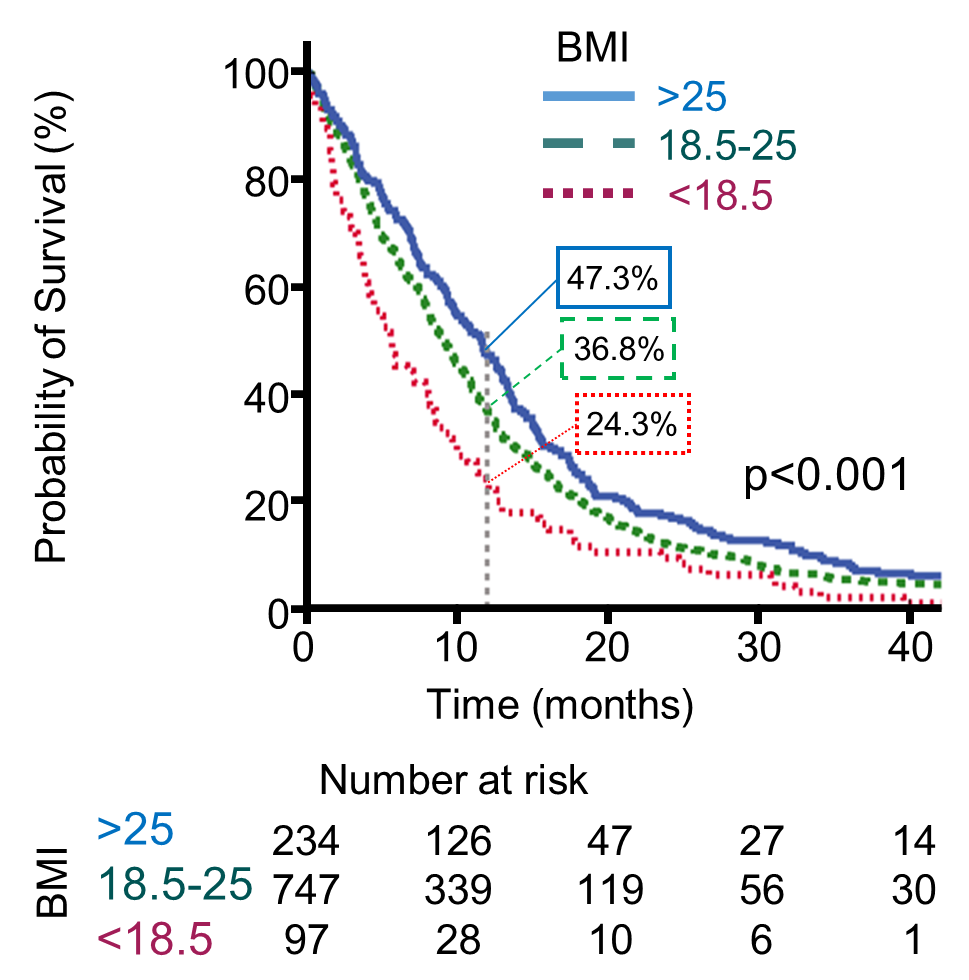


Supplementary Figure 5. Overall survival by composite risk score in subgroup of patients; (A) Metastatic disease; (B) Recurrent disease; (C) Pancreatic cancer; (D) Biliary tract cancer.


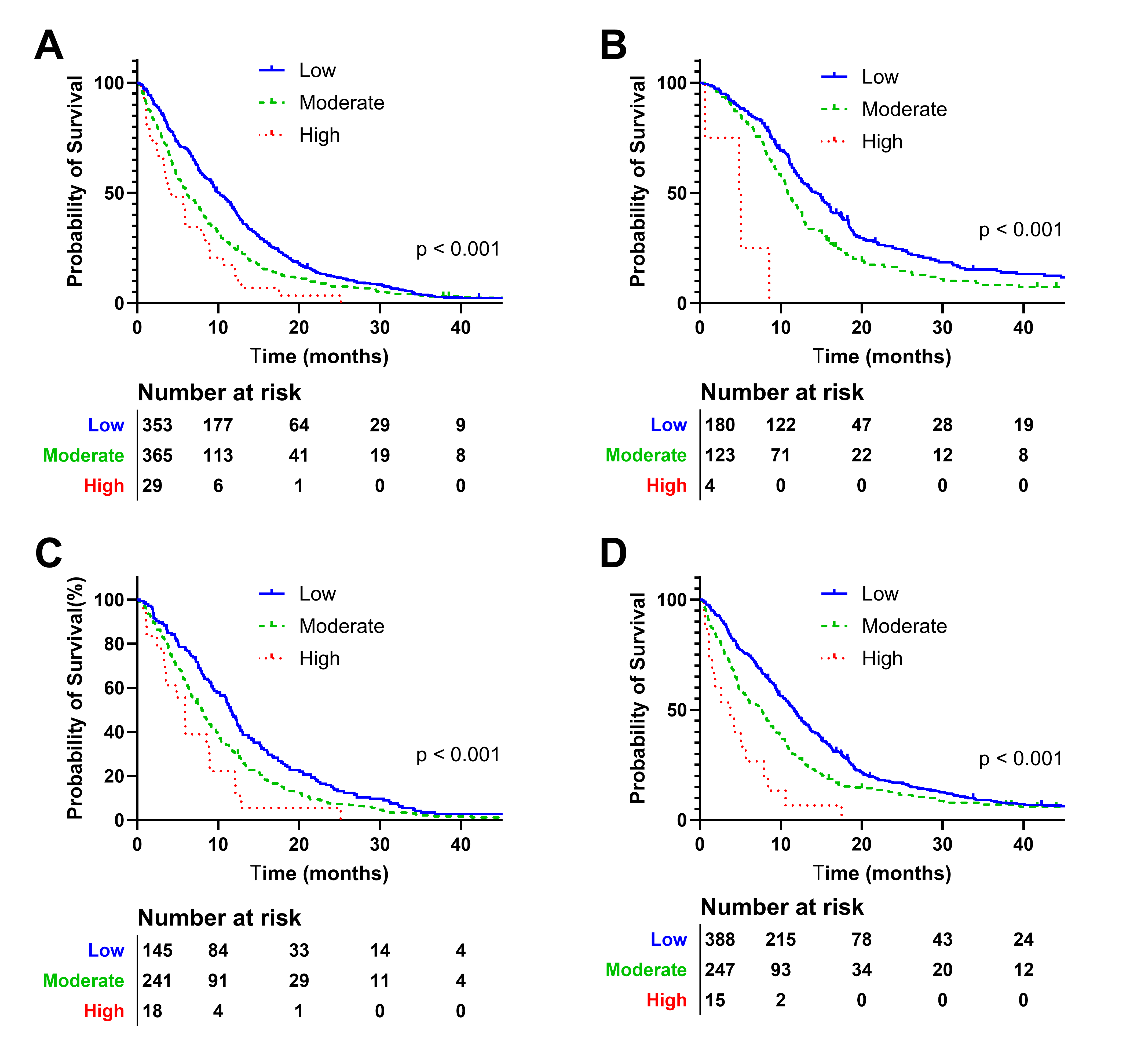


Supplementary Table 1. Logistic regression for prediction of sarcopenia. NRS-2002, nutritional risk screening-2002; ECOG-PS, eastern cooperative oncology group-performance status.

| **Characteristics** | **Variables** | **Logistic regressions analysis** | | |
| --- | --- | --- | --- | --- |
|  |  | **HR** | **95% CI** | ***p*** |
| **Age** | ≥65/<65 | 2.046 | 1.522-2.749 | <0.001 |
| **NRS-2002 score** | ≥ 3/< 3 | 2.932 | 2.169-3.963 | <0.001 |
| **Diabetes Mellitus** | Yes/No | 1.468 | 1.099-1.959 | 0.009 |
| **Hemoglobin (g/dL)** | <13/≥13 | 1.69 | 1.235-2.313 | 0.001 |
| **ECOG-PS** | 2-4/0-1 | 1.477 | 1.097-1.990 | 0.010 |
| **Gender** | Male/Female | 4.354 | 3.171-5.980 | <0.001 |

NRS-2002, nutritional risk screening-2002; ECOG-PS, eastern cooperative oncology group-performance status.

Supplementary Table 2. Univariate and multivariate analyses for progression free survival.

| **Variable** | **Univariate analysis** | | | **Multivariate analysis** | | |
| --- | --- | --- | --- | --- | --- | --- |
|  | **HR** | **95% CI** | ***p*** | **HR** | **95% CI** | ***p*** |
| **BMI (Kg/M^2^)** | | | | | | |
| <18.5/18.5-25.0 | 1.123 | 0.822-1.534 | 0.467 |  |  |  |
| >25.0/18.5-25.0 | 0.858 | 0.706-1.042 | 0.122 |  |  |  |
| **Combining risk group** | | | | | | |
| Moderate/Low | 1.266 | 1.072-1.495 | 0.005 | 1.276 | 1.072-1.519 | 0.006 |
| High/Low | 2.157 | 1.068-4.359 | 0.032 | 2.052 | 1.014-4.154 | 0.046 |
| **WBC (10^3^/μL)** | | | | | | |
| < 4000/≥4000 | 0.911 | 0.638-1.302 | 0.609 |  |  |  |
| **Hemoglobin (g/dL)** | | | | | | |
| <13/≥13 | 1.051 | 0.891-1.240 | 0.555 |  |  |  |
| **ANC (10^3^/μL)** | | | | | | |
| <2/≥2 | 0.974 | 0.609-1.558 | 0.913 |  |  |  |
| **Lymphocyte (10^3^/μL)** | | | | | | |
| <1.5/≥1.5 | 1.274 | 1.081-1.502 | 0.004 | 1.197 | 1.005-1.427 | 0.044 |
| **Total Protein (g/dL)** | | | | | | |
| <6.9/≥6.9 | 0.959 | 0.813-1.131 | 0.620 |  |  |  |
| **CRP (mg/L)** | | | | | | |
| >6.0/≤6.0 | 1.343 | 1.131-1.595 | <0.001 | 1.316 | 1.104-1.567 | 0.002 |
| **CA19-9 (U/mL)** | | | | | | |
| >37.0/≤37.0 | 1.194 | 0.990-1.440 | 0.063 |  |  |  |

BMI, body mass index; WBC, white blood cell; ANC, absolute neutrophil count; CRP, C-reactive protein; CA19-9, carbohydrate antigen 19-9.
